# Exploration of the Effect of Tobacco Smoking on Metabolic Measures in Young People Living with HIV

**DOI:** 10.1155/2014/740545

**Published:** 2014-07-09

**Authors:** Mark L. Rubinstein, D. Robert Harris, Bret J. Rudy, Bill G. Kapogiannis, Grace M. Aldrovandi, Kathleen Mulligan

**Affiliations:** ^1^University of California, San Francisco, San Francisco, CA 94117, USA; ^2^Westat, Rockville, MD 20850, USA; ^3^New York University School of Medicine, New York, NY 10016, USA; ^4^Maternal and Pediatric Infectious Diseases Branch, Eunice Kennedy Shriver National Institute of Child Health and Human Development, NIH, Bethesda, MD 20892, USA; ^5^Children's Hospital Los Angeles, University of Southern California, Los Angeles, CA 90027, USA; ^6^San Francisco General Hospital Division of Endocrinology, Building 30, Room 3501K, 1001 Potrero Avenue, San Francisco, CA 94110, USA

## Abstract

We conducted cross-sectional, multicenter studies in HIV-positive young women and men to assess metabolic and morphologic complications from tobacco smoking in 372 behaviorally infected HIV-positive youth, aged 14–25 years. Measurements included self-reported tobacco use, fasting lipids, glucose, fat distribution, and bone mineral density (BMD; dual-energy X-ray absorptiometry scans). Overall, 144 (38.7%) self-reported smoking tobacco and 69 (47.9%) of these reported smoking greater than five cigarettes per day. Smokers versus nonsmokers had lower mean total cholesterol (146.0 versus 156.1 mg/dL; *P* < 0.01) and lower mean total body fat percent (24.1% versus 27.2%, *P* = 0.03). There was no difference between smokers and nonsmokers in fasting glucose or BMD. There appear to be only minimal effects from tobacco smoking on markers of cardiac risk and bone health in this population of HIV-positive youth. While these smokers may not have had sufficient exposure to tobacco to detect changes in the outcome measures, given the long-term risks associated with smoking and HIV, it is critical that we encourage HIV-positive youth smokers to quit before the deleterious effects become apparent.

## 1. Introduction

Tobacco smoking is still the number one cause of preventable mortality in the United States (US) [1]. Unfortunately, the prevalence of smoking among people living with HIV/AIDS (PLWHA) is much greater than among the general population. In fact, it is estimated that 45% to 74% of adult PLWHA smoke [2–5], compared with 21% of the general population of US adults [6]. Furthermore, as PLWHA live longer, the morbidity and mortality associated with HIV infection has shifted towards heart disease and cancer, both of which are dramatically increased with tobacco smoking [7, 8]. Tobacco smoking is the most important modifiable lifestyle factor contributing to cardiovascular disease among HIV-positive patients [9].

One of the factors associated with the increase in cardiovascular risk among smokers is increased dyslipidemia [10, 11]. Tobacco smoking is also related to cardiovascular risk through its association with insulin resistance and impaired glucose tolerance [12, 13]. Of great importance to PLWHA, all of these cardiovascular effects may be further compounded by the fact that some of the medications used to manage HIV, including protease inhibitors, are associated with increased lipid levels and the development of impaired glucose tolerance and insulin resistance [14].

In addition to its effects on cardiovascular risk factors [10], tobacco smoking is also associated with increased risk of bone fracture in both HIV-negative [15] and HIV-positive populations [16]. The association between tobacco use and bone mineral density (BMD) is influenced by both dose and duration of smoking [17–20]. In fact, tobacco smoking is a risk factor for osteoporotic fractures, independent of body mass and of BMD [20]. Similar to the cardiovascular risk factors discussed above, HIV and some of the medications used to manage it have also been associated with a reduced BMD [21, 22]; thus smoking may compound these effects.

Although studies in adults provide evidence that smoking is associated with markers of accelerated risk for cardiovascular disease and decreased bone mass, it is uncertain whether these abnormalities are found in adolescents and young adult smokers. More importantly, HIV itself, as well as many of the medications used to treat HIV, is also associated with these same metabolic complications in HIV-infected youth and, as such, the combination of smoking and HIV may be even more damaging. This analysis aims to examine the effects of smoking on the lipid, glucose, and bone density profiles of adolescent and young adult smokers with HIV/AIDS, using merged data collected in two studies of the metabolic effects of HIV infection and its therapies in adolescents and young adults.

## 2. Methods

### 2.1. Subjects

Studies were conducted at 18 clinical sites on behalf of the Adolescent Medicine Trials Network for HIV/AIDS Interventions (ATN). Separate cross-sectional surveys were conducted in young women (ATN study 021a [23]) and young men (ATN study 021b [22]) to assess metabolic and morphologic complications in behaviorally HIV-positive youth, aged 14–25 years. Eligible youth were recruited consecutively from the clinic populations. All clinical sites were located in urban areas. The data obtained from these two surveys were combined for this analysis. In the original studies, participants were classified on the basis of current antiretroviral therapy (ART) as follows: antiretroviral naïve (*N* = 190); receiving an ART regimen that contained a nonnucleoside reverse transcriptase inhibitor (NNRTI) but no protease inhibitor (PI) for ≥3 months (*N* = 86); receiving an ART regimen that included a PI but no NNRTI for ≥3 months (*N* = 78); or receiving a non-PI/non-NNRTI containing regimen for ≥3 months (*N* = 18, all female). Those included in the non-PI groups must have had no more than 6 months exposure to a PI in total and none in the preceding year; those in the non-NNRTI groups must have had no more than 6 months total exposure to an NNRTI and none in the preceding year. All participants had acquired HIV infection through risk behavior, were Tanner stage 4 or 5, and had accessible medical and medication histories. Exclusion criteria from both studies included type 1 diabetes mellitus and use of androgens or systemic glucocorticoids. Women were required to have a negative pregnancy test at the time of study unless surgically sterilized.

### 2.2. Informed Consent

The research design and procedures were reviewed and approved by the Institutional Review Boards at each clinical site. Appropriate written informed consent/assent was obtained before enrollment.

### 2.3. Experimental Procedures

Fasting (≥8 hours) blood samples were collected for determination of lipids, glucose, insulin, and high-sensitivity C-reactive protein (hsCRP). Participants then underwent a 2-hour oral glucose tolerance test, with consumption of a 75-gram glucose load and collection of samples for measurement of glucose and insulin. Height and weight were measured following standard protocols. Separate dual-energy X-ray absorptiometry (DXA) scans of the left hip, spine, and whole body were performed with central analysis at Tufts University by readers who were blinded to ART regimen. Machine-generated Z-scores (using sex-specific standard deviations adjusted for race/ethnicity and age) for spine (L1-L4) and hip BMD were used. Z-scores for total body bone mineral content (BMC) were calculated using norms developed at Baylor University [24]. Total and regional fat and lean body mass (LBM) were obtained from the whole-body DXA scans and normalized for height squared.

All participants underwent detailed medical and health histories, including current and previous drug use. Dietary intake, alcohol use, and exercise and smoking habits were assessed using the Block Food Frequency Questionnaire (NutritionQuest, Berkeley, CA). Current tobacco use was queried in the Food Frequency Questionnaire as follows: the participant was first asked “do you smoke cigarettes now”; if the answer was “yes,” the questionnaire then asked “on average about how many cigarettes a day do you smoke now,” with answers categorized as “1–5, 6–14, 15–24, 25–34, and 35 or more.”

### 2.4. Laboratory Analyses

HIV-1 RNA levels (Roche AMPLICOR v1.5 assay) and CD4 T-cell counts were measured locally at each site. All other laboratory samples were batched at the end of each study and analyzed at Quest Diagnostics, Baltimore, MD, and Quest Diagnostics Nichols Institute, Chantilly, VA. Total and high-density lipoprotein (HDL) cholesterol and triglycerides were measured by enzymatic techniques, and low density lipoprotein (LDL) cholesterol was calculated in those with triglyceride levels <400 mg/dL [25]. Specimens for glucose determination, which were collected on sodium fluoride/potassium oxalate, were assessed by the hexokinase technique. Serum insulin was measured by immunoassay and hsCRP was measured by a particle-enhanced immunonephelometric assay.

### 2.5. Data Analyses

Data collected from all HIV-positive participants in the parent studies were included in this analysis. The distribution of continuous measures according to smoking status was examined on the basis of means, standard deviations (SD), and medians, with *P* values provided to assess the significance of the associations with smoking status using Student's two-sample *t*-test. Frequencies and proportions are reported for categorical measures according to smoking status, with *P* values obtained using Fisher's exact test. In addition to examining associations of smoking status with metabolic and body habitus measures, associations of smoking status with other lifestyle factors, such as use of alcohol and recreational drugs, were explored.

Generalized linear regression modeling was used to examine the association of cigarette smoking with study outcomes, adjusting for covariates selected for inclusion in the model using a stepwise forward selection approach. Candidate covariates, including antiretroviral therapy type, were screened for possible inclusion in the modeling on the basis of their bivariable associations with smoking status, with an alpha level of ≤0.10 used for selection. Covariates whose distributions would pose problems for the modeling due to missing data or small cell sizes in the bivariable analyses were excluded from consideration. Covariates that are traditionally considered for adjustment (i.e., age, sex, race, and height) were included in the modeling regardless of the significance of their bivariable associations with smoking status. HIV-specific candidate covariates included HIV RNA, CD4 measures, CDC disease classification, and the type of ART regimen subjects who were receiving at the time of study enrollment, as well as whether or not their current ART regimen included ritonavir, a PI that has been widely associated with lipid abnormalities [14]. The modeling also considered individual substance use, including use of alcohol, cannabinoids (marijuana or hash), and stimulants (cocaine, crack, amphetamine sulphate, methamphetamine, or other stimulants). The possibility of a dose-response relationship between the average number of cigarettes smoked per day and the study outcomes was also explored in the modeling by separating tobacco users into those who reported smoking ≤5 cigarettes/day and those reporting >5 cigarettes/day. Data from one participant who was using a statin were excluded from the modeling analyses.

## 3. Results

In this population of HIV-positive participants, 144 (38.7%) self-reported smoking tobacco, and 69 (47.9%) of these reported smoking greater than 5 cigarettes per day (CPD). Demographic and HIV disease-related characteristics of the participants are presented in Table 1. Reported time since first HIV-positive diagnosis did not differ significantly between smokers and nonsmokers (*P* = 0.3). However, smokers had a significantly higher mean log_10_ viral load (HIV RNA level), and a smaller proportion of the smokers had HIV RNA ≤400 copies/mL (*P* ≤ 0.02 for both measures). Mean CD4 T lymphocyte counts did not differ significantly between smokers and nonsmokers (*P* = 0.18). There were no significant differences between groups in use of different classes of antiretroviral therapies (*P* = 0.17).

### 3.1. Lifestyle

Use of alcohol, cannabinoids, cocaine, and methamphetamines was reported significantly more frequently by smokers than nonsmokers (*P* ≤ 0.002). Less than half of smokers and nonsmokers reported regularly exercising (42.4 versus 43.0%, resp.; *P* = 0.91).

### 3.2. Fasting Lipids

Mean total cholesterol was significantly lower among smokers than nonsmokers (146.0 versus 156.1 mg/dL; *P* = 0.003) (Table 2). These results remained significant after adjusting for possible confounders (*P* = 0.009). HDL cholesterol tended to be lower in smokers, but the difference did not achieve statistical significance in the unadjusted (*P* = 0.07) or adjusted (*P* = 0.09) analyses. The mean level of LDL cholesterol was similar between smokers and nonsmokers (*P* = 0.52). Mean non-HDL cholesterol levels were also significantly lower in smokers than nonsmokers (107.2 versus 114.0 mg/dL; *P* = 0.03). These results were not significant after adjusting for relevant confounders.

### 3.3. Fasting Glucose/Insulin

Mean fasting glucose levels were similar between smokers and nonsmokers. The 2-hour glucose was lower in smokers, although only of borderline significance in the adjusted model (87.8 versus 93.0 mg/dL; *P* = 0.08) (Table 2). Similarly, there was no difference in mean fasting insulin or 2-hour insulin levels for smokers compared to nonsmokers (*P* > 0.1).

### 3.4. Body Composition/Bone Mass

Adjusting for covariates, mean body mass index (BMI) was similar in smokers and nonsmokers (25.0 versus 25.8 kg/m^2^; *P* = 0.24). However, differences in body composition were noted. Mean total body fat percent was significantly lower in smokers (24.1% versus 27.2%; *P* = 0.03); this difference persisted with adjustment for relevant confounders (*P* = 0.03). Most of this difference appears to be related to lower extremity fat where smokers had 2.4 compared with 2.9 kg/m^2^ in nonsmokers (*P* = 0.04 in unadjusted and adjusted analyses). Considering the number of cigarettes smoked per day (Table 3), the difference in mean total body fat percent among smoking categories was only of borderline significance (*P* = 0.06), yet pairwise comparisons indicated that the mean level was significantly lower among those who smoked an average of 5 or fewer cigarettes per day than among nonsmokers (23.0% versus 27.2%; *P* < 0.05). Lean body mass did not differ between smokers and nonsmokers in either the unadjusted or adjusted analyses (Table 2). There were no differences between smokers and nonsmokers in any measure of BMD or BMC (*P* > 0.1, data not shown).

## 4. Discussion

We examined the effects of tobacco smoking on markers of cardiovascular and bone health among a sample of behaviorally infected young HIV-positive smokers and nonsmokers. Although total cholesterol was lower among smokers, much of this difference may have been accounted for by the lowering of HDL cholesterol. In fact, similar to findings in adult tobacco smokers [26, 27], we found a trend toward lower HDL cholesterol among this group of young smokers, albeit of borderline significance. Roughly half of these smokers smoked fewer than 5 CPD and, given their age, had likely been smoking for fewer years than many of those included in the adult studies, which may account for the weak association of smoking with HDL cholesterol in our study population.

This group of young smokers had a lower total body fat percentage and less leg fat than nonsmokers. Studies in adults typically report lower BMI in smokers and increases in weight and fat with smoking cessation [28–30]. Some studies in adults have suggested that smoking is associated with a pattern of central adiposity, based on higher waist circumferences and lower hip circumferences [28, 29]. Using DXA, a more sensitive measure of fat distribution, we observed no significant difference between smokers and nonsmokers in central (trunk) fat but significantly lower leg fat. These observations could reflect a relative preservation of central fat among HIV-positive young smokers. DXA cannot distinguish between intra-abdominal and subcutaneous fat in the trunk region, so we cannot exclude the possibility that smokers and nonsmokers may differ with regard to intra-abdominal fat.

Unlike findings reported in adult smokers [12, 13], there did not appear to be any difference in glucose tolerance or insulin resistance in these adolescent and young adult smokers, even after adjusting for possible confounders. We also did not find appreciable differences in bone mass between smokers and nonsmokers. As with the lipid findings, it is possible that these youth had not had sufficient exposure to tobacco smoking to adversely affect their glucose and insulin profiles or their bone density.

Taken together, our findings suggest that tobacco smoking has had little to no effect on the metabolic and bone density profiles of these youth. Whether or not this is due to the lower cumulative exposure to tobacco smoke compared with the older smokers reported in the adult studies is uncertain. Clearly longitudinal studies with greater exposure to tobacco are required to examine these parameters over time. However, given the known risks of tobacco smoking and the combined effects of HIV and smoking observed in studies in adults, there are notable implications for secondary prevention of such long-term untoward effects among this young population facing a long life with HIV, its therapies, and associated comorbidities. Our results provide an opportunity to capitalize on the potential long-term benefits that could be achieved by targeting HIV-infected smoking youth with efficacious smoking cessation interventions, early in this window in which there is little evidence that any appreciable harm has already occurred. Offering HIV-infected youth information on the combined risk from smoking and HIV, along with noting the fact that there is still time to make lifestyle changes (i.e., quit smoking) before significant damage is done, may prove a powerful motivator.

*Limitations.* A potential limitation of our study is that we relied on self-reported smoking status. However, prior data from a similar sample of HIV-positive adolescents showed a high concordance between self-report and cotinine, a biomarker of tobacco exposure [31]. We did not collect data on years from initiation of cigarette smoking and so we cannot make inferences about the duration of smoking on the metabolic profile of HIV-positive tobacco smokers. As such, the apparent lack of effects of tobacco smoking on the metabolic profiles of these participants may reflect a relatively limited exposure to tobacco. It is also possible that some of the differences observed would become more pronounced over time. All clinical sites were located in large urban areas, so these results may not be representative of HIV-infected youth in general. Finally, although we did not have data on actual CPD smoked, but rather a less precise measure of smoking categories (i.e., ≤5 CPD, 6–14 CPD, 15–24 CPD, 25–34 CPD, and ≥35 CPD), the trends observed from using the smoking categories suggest that there is little dose-response effect from tobacco on these young smokers.

## 5. Conclusion

In summary, there appear to be only minimal effects from tobacco smoking on markers of cardiac risk and bone health in this population of HIV-positive youth. It may be that these HIV-positive smokers had not had sufficient exposure to tobacco to detect significant changes. However, given the long-term risks associated with smoking and the likely compounding effects of HIV and treatments for HIV, it is critical that we find effective targeted interventions to encourage HIV-positive youth smokers to quit smoking before the deleterious effects become apparent.

## Figures and Tables

**Table 1:** Demographics of HIV-positive youth smokers and nonsmokers.

Variable	Smokers (*n* = 144)	Nonsmokers (*n* = 228)	*P* value^1^
Age in years: mean (SD)	21.6 (2.0)	20.8 (2.3)	**<0.001**
Race: *n* (%)			
Black/African American	89 (62.7)	168 (74.3)	**0.05**
White	19 (13.4)	17 (7.5)	
Other/mixed race	34 (23.9)	41 (18.1)	
Hispanic ethnicity: *n* (%)	38 (26.4)	48 (21.1)	0.26
Years since HIV-positive diagnosis: mean (SD)	2.5 (2.2)	2.2 (2.0)	0.30
Current log_10_ viral load (copies/mL): mean (SD)	3.4 (1.0)	3.2 (1.1)	**0.02**
Current viral load ≤400 copies/mL: *n* (%)	49 (34.5)	109 (48.2)	**0.01**
Current CD4 count (cells/mm^3^): mean (SD)	495 (273)	536 (319)	0.18
Current antiretroviral use by drug class: *n* (%)			
ART-naive	84 (58.3)	106 (46.5)	0.17
NNRTI, no PI	27 (18.8)	59 (25.9)	
PI, no NNRTI	27 (18.8)	51 (22.4)	
Non-PI, non-NNRTI^2^	6 (4.2)	12 (5.3)	
Use alcohol: *n* (%)	123 (85.4)	136 (59.6)	**<0.001**
Use marijuana, hash: *n* (%)	120 (83.3)	95 (41.7)	**<0.001**
Use crack: *n* (%)	6 (4.2)	3 (1.3)	0.09
Use cocaine: *n* (%)	43 (29.9)	18 (7.9)	**<0.001**
Use methamphetamine: *n* (%)	20 (13.9)	10 (4.4)	**0.002**
Exercise regularly: *n* (%)	61 (42.4)	98 (43.0)	0.91

SD = standard deviation.

^1^ *P* values obtained from Student's *t*-test for continuous measures and Fisher's exact test for categorical measures.

^2^This antiretroviral drug class was used only in the study of females.

**Table 2:** Metabolic factors and smoking status among HIV-positive youth.

Metabolic factor	Smokers Mean (SD) Median	Nonsmokers Mean (SD) Median	Unadjusted *P* value^1^	Adjusted *P* value^2^
Total cholesterol (mg/dL)	146.0 (29.5) 145.5	156.1 (33.4) 153.0	**0.003**	**0.009**
HDL cholesterol (mg/dL)	61.9 (34.7) 45.5	68.8 (35.6) 60.5	0.07	0.09
LDL cholesterol (mg/dL)	64.1 (28.8) 60.0	66.9 (31.6) 57.0	0.39	0.52
Non-HDL cholesterol (mg/dL)	107.2 (27.4) 105.0	114.0 (33.0) 110.0	**0.03**	0.08
Triglycerides (mg/dL)	100.0 (53.3) 90.0	98.8 (63.3) 82.0	0.85	0.83
Fasting glucose (mg/dL)	88.5 (7.5) 88.0	88.2 (8.1) 88.0	0.71	0.77
2-hour glucose (mg/dL)	87.8 (26.5) 85.0	93.0 (24.0) 92.0	0.05	0.08
Fasting insulin (*µ*IU/mL)	9.4 (7.4) 8.0	10.6 (11.3) 8.0	0.23	0.31
2-hour insulin (*µ*IU/mL)	42.4 (46.5) 29.0	49.6 (46.4) 39.0	0.16	0.16
hsCRP (mg/dL)	3.50 (6.52) 1.00	2.96 (5.91) 1.00	0.41	0.40
BMI (kg/m^2^)	25.0 (6.5) 23.5	25.8 (6.2) 24.1	0.22	0.24
Total body fat percent	24.1 (13.1) 20.7	27.2 (13.2) 25.6	**0.03**	**0.03**
Total body fat (kg/m^2^)	6.4 (5.1) 4.5	7.3 (5.0) 5.6	0.09	0.10
Trunk fat (kg/m^2^)	3.0 (2.5) 2.1	3.4 (2.5) 2.5	0.15	0.18
Lower extremity (leg) fat (kg/m^2^)	2.4 (2.1) 1.7	2.9 (2.1) 2.4	**0.04**	**0.04**
Total lean body mass (kg/m^2^)	17.3 (2.5) 17.0	17.3 (2.5) 17.1	0.83	0.83

SD = standard deviation.

^1^ *P* values obtained from Student's *t*-test.

^2^ *P* values obtained from generalized linear regression modeling; smoking status, race, height, gender, and age at enrollment were included in all of the models, regardless of their significance, while current antiretroviral regimen and current use of marijuana, stimulants, or alcohol were included only if they contributed significantly to the model.

**Table 3:** Association of average number of cigarettes smoked per day (CPD) with metabolic markers among HIV-positive youth.

Outcome measure	≤5 CPD Mean (SD) Median	>5 CPD Mean (SD) Median	Nonsmokers Mean (SD) Median	*P* value^1^
HDL cholesterol (mg/dL)	60.5 (33.9) 45.0	63.1 (35.8) 50.0	68.7 (35.6) 60.0	0.18
LDL cholesterol (mg/dL)	65.0 (28.1) 61.0	63.1 (29.6) 59.0	67.0 (31.6) 57.0	0.63
Non-HDL cholesterol (mg/dL)	105.7 (28.6) 105.0	108.5 (26.5) 104.0	114.0 (33.1) 110.0	0.10
Triglycerides (mg/dL)	97.4 (50.7) 85.0	102.5 (56.8) 92.0	98.8 (63.5) 82.0	0.87
Fasting glucose (mg/dL)	88.4 (8.0) 88.0	88.9 (6.8) 88.0	88.2 (8.1) 88.0	0.84
2-hour glucose (mg/dL)	85.8 (26.1) 83.5	90.3 (27.2) 87.0	93.1 (24.0) 92.0	0.10
Fasting insulin (*µ*IU/mL)	9.0 (6.5) 8.0	9.8 (8.4) 8.0	10.6 (11.3) 8.0	0.47
2-hour insulin (*µ*IU/mL)	46.9 (57.0) 29.5	38.5 (33.1) 26.0	49.8 (46.5) 39.0	0.22
hsCRP (mg/dL)	2.8 (5.9) 0.9	4.3 (7.2) 1.5	3.0 (5.9) 1.0	0.25
BMI (kg/m^2^)	24.6 (6.5) 22.9	25.5 (6.6) 23.9	25.8 (6.2) 24.1	0.37
Total body fat percent	23.0 (12.6)∗ 18.2	25.3 (13.7) 22.8	27.2 (13.2) 25.4	0.06
Total body fat (kg/m^2^)	6.0 (4.7) 4.0	6.9 (5.5) 5.3	7.3 (5.0) 5.6	0.15
Trunk fat (kg/m^2^)	2.7 (2.3) 1.8	3.3 (2.7) 2.5	3.4 (2.5) 2.5	0.13
Lower extremity fat (kg/m^2^)	2.4 (2.0) 1.6	2.5 (2.3) 1.8	2.9 (2.1) 2.4	0.12
Total body lean (kg/m^2^)	17.3 (2.4) 17.1	17.4 (2.7) 17.0	17.3 (2.5) 17.1	0.93

SD = standard deviation.

^1^ *P* values obtained from generalized linear regression modeling (GLM).

∗Significantly different compared to nonsmokers (*P* < 0.05).
